# 3-[2-(Triphenyl­phosphanyl­idene)acet­yl]-2*H*-chromen-2-one

**DOI:** 10.1107/S160053681300127X

**Published:** 2013-01-19

**Authors:** Muhammad Taha, Nor Hadiani Ismail, Ahmad Nazif Aziza, Syed Adnan Ali Shah, Sammer Yousuf

**Affiliations:** a Atta-ur-Rahman Institute for Natural Product Discovery (RiND), Universiti Teknologi MARA (UiTM), Puncak Alam Campus, 42300 Bandar Puncak Alam, Selangor D. E., Malaysia; b Faculty of Applied Science, Universiti Teknologi MARA (UiTM), 40450 Shah Alam, Selangor D. E., Malaysia; c Faculty of Applied Sciences, Universiti Teknologi MARA (UiTM), 40000 Shah Alam, Selangor D. E., Malaysia; d Department of Chemical Sciences, Faculty of Science and Technology, University Malaysia Terengganu, 21030 Kuala Terengganu, Malaysia; e Department of Pharmacology and Chemistry, Faculty of Pharmacy, Universiti Teknologi MARA (UiMT) Puncak Alam Campus, 42300 Puncak Alam, Selangor D. E., Malaysia; f H.E.J. Research Institute of Chemistry, International Center for Chemical and Biological Sciences, University of Karachi, Karachi 75270, Pakistan

## Abstract

In the title compound, C_29_H_21_O_3_P, a coumarin-substitued ylid, the P atom is linked to three benzene rings and a planar coumarin moiety *via* a methyl­enecarbonyl group. The bond lengths in the P=C–C=O fragment clearly indicate a delocalized system involving the olefinic and carbonyl bonds. The mol­ecular structure is stabilized by an intra­molecular C—H⋯O inter­action that results in an *S*7 graph-set ring motif. In the crystal, mol­ecules are linked into a three-dimensional framework by C—H⋯O hydrogen bonds.

## Related literature

For applications and biological activity of coumarin, see: Kabak *et al.* (1999 ▶); El-Ansary *et al.* (1992 ▶); Czerpack & Skolska (1982 ▶); Reddy & Somayojulu (1981 ▶); Jund *et al.* (1971 ▶). For the crystal structure of a related compound, see: Schobert *et al.* (2000 ▶).
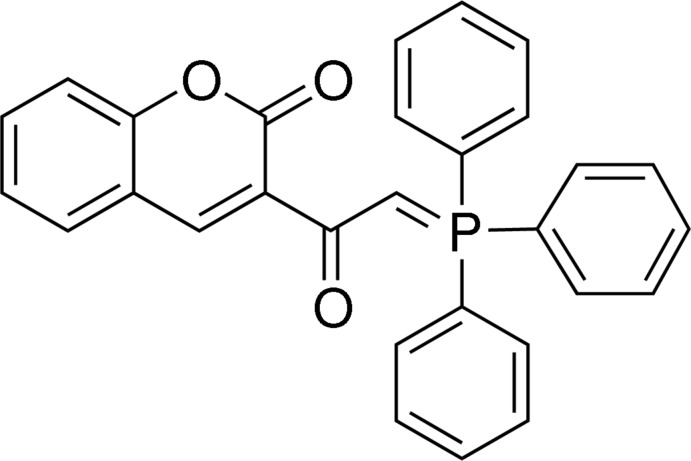


## Experimental

### 

#### Crystal data

C_29_H_21_O_3_P
*M* *_r_* = 448.43
Triclinic, 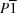

*a* = 9.7837 (12) Å
*b* = 10.3917 (14) Å
*c* = 12.2925 (17) Å
α = 108.669 (4)°
β = 104.484 (4)°
γ = 99.746 (4)°
*V* = 1103.2 (3) Å^3^
*Z* = 2
Mo *K*α radiation
μ = 0.16 mm^−1^
*T* = 100 K
0.46 × 0.41 × 0.34 mm

#### Data collection

Bruker APEXII CCD diffractometer
Absorption correction: multi-scan (*SADABS*; Bruker, 2000 ▶) *T*_min_ = 0.932, *T*_max_ = 0.949
36693 measured reflections
4102 independent reflections
3716 reflections with *I* > 2σ(*I*)
*R*_int_ = 0.045

#### Refinement

*R*[*F* ^2^ > 2σ(*F* ^2^)] = 0.035
*wR*(*F* ^2^) = 0.093
*S* = 1.07
4102 reflections
299 parameters
H-atom parameters constrained
Δρ_max_ = 0.32 e Å^−3^
Δρ_min_ = −0.40 e Å^−3^

### 

Data collection: *APEX2* (Bruker, 2000 ▶); cell refinement: *SAINT* (Bruker, 2000 ▶); data reduction: *SAINT*; program(s) used to solve structure: *SHELXS97* (Sheldrick, 2008 ▶); program(s) used to refine structure: *SHELXL97* (Sheldrick, 2008 ▶); molecular graphics: *SHELXTL* (Sheldrick, 2008 ▶); software used to prepare material for publication: *SHELXTL*.

## Supplementary Material

Crystal structure: contains datablock(s) global, I. DOI: 10.1107/S160053681300127X/pv2616sup1.cif

Structure factors: contains datablock(s) I. DOI: 10.1107/S160053681300127X/pv2616Isup2.hkl

Supplementary material file. DOI: 10.1107/S160053681300127X/pv2616Isup3.cml

Additional supplementary materials:  crystallographic information; 3D view; checkCIF report

## Figures and Tables

**Table 1:** Hydrogen-bond geometry (Å, °)

*D*—H⋯*A*	*D*—H	H⋯*A*	*D*⋯*A*	*D*—H⋯*A*
C2—H2*A*⋯O2^i^	0.95	2.45	3.378 (2)	166
C7—H7*A*⋯O3^ii^	0.95	2.28	3.171 (2)	156
C22—H22*A*⋯O2^iii^	0.95	2.48	3.398 (2)	163
C25—H25*A*⋯O3	0.95	2.31	3.168 (2)	150
C28—H28*A*⋯O1^iv^	0.95	2.54	3.281 (2)	135

Symmetry codes: (i) 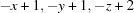; (ii) 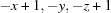; (iii) 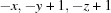; (iv) 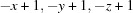.

**Fig. 1.**
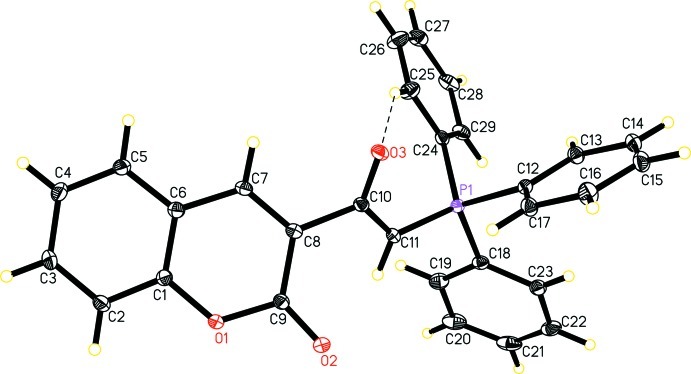
The molecular structure of the title compound with the atom numbering scheme. Displacement ellipsoids are drawn at the 30% probability level. H atoms are presented as small spheres of arbitrary radius.

**Fig. 2.**
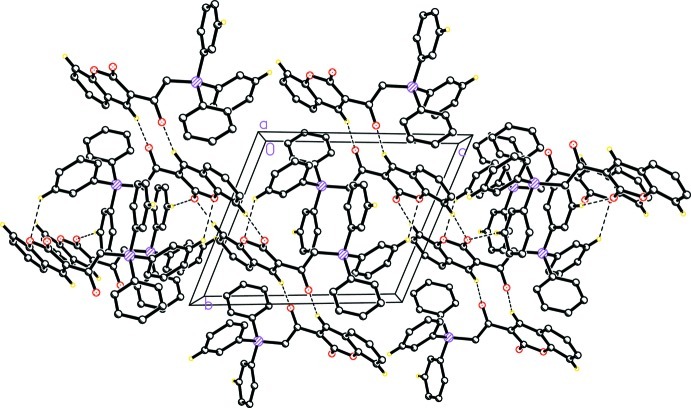
A view of the C—-H···O hydrogen bonds (dotted lines) in the crystal structure of the title compound. H atoms non-participating in hydrogen- bonding were omitted for clarity.

## supplementary crystallographic information

### Comment

The chromone chemistry continues to draw considerable interest of synthetic organic and medicinal chemists (Kabak *et al.*, 1999). Chromones are more widely distributed in nature, especially in the plant kingdom, and exhibit low toxicity along with a wide spectrum of useful biological activities including antifungal, antiviral, antitublin, anti-inflammatory antiulcer and antihypertensive and immune-stimulating properties (El-Ansary *et al.*, 1992; Czerpack & Skolska, 1982; Reddy & Somayojulu, 1981; Jund *et al.*, 1971). The title compound is a coumarin substitued ylid synthesized as a part of our ongoing resaerch to study biological activities of this medicinally important class of compounds.

The bond distances and angles in the title compound (Fig. 1) agree very well with the corresponding bond distances and angles reported in a closely related compound (Schobert *et al.*, 2000). In the title molecule, the central phosphorus atom adopts a tetrahedral geometry and is linked to three benzene rings and a planner coumarin moiety (maximum deviation of 0.005 (2) Å for C1 atom) *via* methylene carbonyl group. The bond lengths P1–C11 (1.7237 (14) Å) and C10–C11 (1.395 (2) Å), deviating from typical P═C (1.67 Å) and C–C (1.50 Å) support the congugation of double bond with that of carbonyl group *via* keto enol tautomerization. The geomatry of the molecule is stablizied by an intramolecular C25—H25A···O3 hydrogen bonding interaction. The crystal structure is stabilized by intermolecular C2—H2A···O2, C7—H7A···O3, C22—H22A···O2 and C28—H28A···O1 interactions forming a three-dimensional network (Table 1 and Fig. 2).

### Experimental

The title compound was synthesized in two steps. In the first step, 3-((triphenylphosphinyl) acetyl)coumarin bromide was synthesized by treating 3-(bromoacetyl)coumarin (2 mmol, 0.534 g) in 10 ml of CH_2_Cl_2_ and triphenylphosphine (2 mmol, 0.524 g). The mixture was stirred for 3 hrs at room temperature. The solvent was evaporated and washed with diethyl ether, to obtain a yellow crystalline solid (96% yield, 1.14 g). In the next step 3-((triphenylphosphinyl) acetyl)coumarin bromide (1 mmol, 0.528 g) was dissolved in ethanol (10 ml), treated dropwise with potassium carbonate (1 mmol, 0.1 g) in 5 ml of H_2_O, stirred for 1.5 h at room temperature, diluted with 40 ml of H_2_O, and extracted with 4 × 10 ml of EtOAc. The combined organic phases were dried over MgSO_4_, filtered, and evaporated under reduced pressure to give the title compound as a yellow crystalline solid (90% yield, 0.403 g). Mp: 388–390 K.

### Refinement

H atoms on were positioned geometrically with C–H = 0.95 Å and constrained to ride on their parent atoms with *U*_iso_(H) = 1.2*U*_eq_(C).

### Crystal data

**Table:**

C_29_H_21_O_3_P	*Z* = 2
*M**_r_* = 448.43	*F*(000) = 468
Triclinic, *P*1	*D*_x_ = 1.350 Mg m^−^^3^
Hall symbol: -P 1	Mo *K*α radiation, λ = 0.71073 Å
*a* = 9.7837 (12) Å	Cell parameters from 7459 reflections
*b* = 10.3917 (14) Å	θ = 3.2–26.4°
*c* = 12.2925 (17) Å	µ = 0.16 mm^−^^1^
α = 108.669 (4)°	*T* = 100 K
β = 104.484 (4)°	Block, yellow
γ = 99.746 (4)°	0.46 × 0.41 × 0.34 mm
*V* = 1103.2 (3) Å^3^	

### Data collection

**Table:**

Bruker APEXII CCD diffractometer	4102 independent reflections
Radiation source: fine-focus sealed tube	3716 reflections with *I* > 2σ(*I*)
Graphite monochromator	*R*_int_ = 0.045
ω scan	θ_max_ = 25.5°, θ_min_ = 3.2°
Absorption correction: multi-scan (*SADABS*; Bruker, 2000)	*h* = −11→11
*T*_min_ = 0.932, *T*_max_ = 0.949	*k* = −12→12
36693 measured reflections	*l* = −14→14

### Refinement

**Table:**

Refinement on *F*^2^	Secondary atom site location: difference Fourier map
Least-squares matrix: full	Hydrogen site location: inferred from neighbouring sites
*R*[*F*^2^ > 2σ(*F*^2^)] = 0.035	H-atom parameters constrained
*wR*(*F*^2^) = 0.093	*w* = 1/[σ^2^(*F*_o_^2^) + (0.0408*P*)^2^ + 0.6733*P*] where *P* = (*F*_o_^2^ + 2*F*_c_^2^)/3
*S* = 1.07	(Δ/σ)_max_ < 0.001
4102 reflections	Δρ_max_ = 0.32 e Å^−^^3^
299 parameters	Δρ_min_ = −0.40 e Å^−^^3^
0 restraints	Extinction correction: *SHELXL97* (Sheldrick, 2008), Fc^*^=kFc[1+0.001xFc^2^λ^3^/sin(2θ)]^-1/4^
Primary atom site location: structure-invariant direct methods	Extinction coefficient: 0.041 (3)

### Special details

**Table:**

Geometry. All e.s.d.'s (except the e.s.d. in the dihedral angle between two l.s. planes) are estimated using the full covariance matrix. The cell e.s.d.'s are taken into account individually in the estimation of e.s.d.'s in distances, angles and torsion angles; correlations between e.s.d.'s in cell parameters are only used when they are defined by crystal symmetry. An approximate (isotropic) treatment of cell e.s.d.'s is used for estimating e.s.d.'s involving l.s. planes.
Refinement. Refinement of *F*^2^ against ALL reflections. The weighted *R*-factor *wR* and goodness of fit *S* are based on *F*^2^, conventional *R*-factors *R* are based on *F*, with *F* set to zero for negative *F*^2^. The threshold expression of *F*^2^ > σ(*F*^2^) is used only for calculating *R*-factors(gt) *etc*. and is not relevant to the choice of reflections for refinement. *R*-factors based on *F*^2^ are statistically about twice as large as those based on *F*, and *R*- factors based on ALL data will be even larger.

### Fractional atomic coordinates and isotropic or equivalent isotropic displacement parameters (Å^2^)

**Table:**

	*x*	*y*	*z*	*U*_iso_*/*U*_eq_	
P1	0.22033 (4)	0.28713 (4)	0.37638 (3)	0.01546 (12)	
O1	0.54288 (11)	0.37113 (10)	0.89176 (9)	0.0192 (2)	
O2	0.32695 (11)	0.37143 (11)	0.78335 (9)	0.0227 (2)	
O3	0.32489 (11)	0.06590 (10)	0.47387 (9)	0.0213 (2)	
C1	0.67160 (16)	0.33179 (15)	0.90304 (13)	0.0178 (3)	
C2	0.78228 (17)	0.39917 (16)	1.01425 (13)	0.0222 (3)	
H2A	0.7684	0.4680	1.0801	0.027*	
C3	0.91346 (17)	0.36328 (17)	1.02647 (14)	0.0251 (3)	
H3A	0.9915	0.4093	1.1014	0.030*	
C4	0.93292 (18)	0.26033 (17)	0.93023 (14)	0.0264 (3)	
H4A	1.0236	0.2366	0.9403	0.032*	
C5	0.82116 (17)	0.19312 (16)	0.82082 (14)	0.0235 (3)	
H5A	0.8346	0.1223	0.7560	0.028*	
C6	0.68754 (16)	0.22893 (15)	0.80479 (13)	0.0187 (3)	
C7	0.57153 (16)	0.17586 (15)	0.69048 (12)	0.0183 (3)	
H7A	0.5774	0.1018	0.6237	0.022*	
C8	0.45432 (15)	0.22872 (14)	0.67566 (12)	0.0166 (3)	
C9	0.43323 (15)	0.32761 (14)	0.78122 (12)	0.0171 (3)	
C10	0.35227 (15)	0.19030 (15)	0.54841 (12)	0.0168 (3)	
C11	0.30703 (15)	0.30007 (15)	0.52151 (12)	0.0174 (3)	
H11A	0.3250	0.3869	0.5869	0.021*	
C12	0.05596 (15)	0.14349 (15)	0.29385 (13)	0.0179 (3)	
C13	−0.01368 (16)	0.10991 (16)	0.17037 (13)	0.0221 (3)	
H13A	0.0293	0.1585	0.1284	0.027*	
C14	−0.14561 (17)	0.00551 (17)	0.10911 (13)	0.0244 (3)	
H14A	−0.1942	−0.0159	0.0256	0.029*	
C15	−0.20609 (17)	−0.06731 (17)	0.16996 (14)	0.0275 (4)	
H15A	−0.2963	−0.1388	0.1281	0.033*	
C16	−0.13548 (18)	−0.03636 (18)	0.29201 (15)	0.0303 (4)	
H16A	−0.1765	−0.0880	0.3329	0.036*	
C17	−0.00522 (16)	0.06981 (17)	0.35421 (13)	0.0233 (3)	
H17A	0.0421	0.0921	0.4381	0.028*	
C18	0.17083 (17)	0.44966 (15)	0.39085 (13)	0.0198 (3)	
C19	0.28216 (19)	0.57658 (16)	0.45100 (15)	0.0274 (3)	
H19A	0.3809	0.5754	0.4821	0.033*	
C20	0.2484 (2)	0.70386 (18)	0.46519 (16)	0.0346 (4)	
H20A	0.3235	0.7902	0.5075	0.042*	
C21	0.1052 (2)	0.70496 (19)	0.41764 (16)	0.0370 (4)	
H21A	0.0830	0.7922	0.4245	0.044*	
C22	−0.0057 (2)	0.5810 (2)	0.36029 (15)	0.0357 (4)	
H22A	−0.1041	0.5833	0.3295	0.043*	
C23	0.02628 (18)	0.45223 (18)	0.34736 (14)	0.0260 (3)	
H23A	−0.0503	0.3668	0.3090	0.031*	
C24	0.33364 (15)	0.26597 (15)	0.27860 (12)	0.0181 (3)	
C25	0.41259 (18)	0.16625 (18)	0.27691 (15)	0.0278 (4)	
H25A	0.4064	0.1125	0.3260	0.033*	
C26	0.5006 (2)	0.1454 (2)	0.20336 (17)	0.0350 (4)	
H26A	0.5549	0.0777	0.2028	0.042*	
C27	0.50937 (18)	0.22260 (18)	0.13103 (15)	0.0303 (4)	
H27A	0.5701	0.2083	0.0813	0.036*	
C28	0.42996 (19)	0.32023 (16)	0.13125 (14)	0.0287 (4)	
H28A	0.4352	0.3724	0.0808	0.034*	
C29	0.34227 (17)	0.34276 (16)	0.20488 (13)	0.0238 (3)	
H29A	0.2882	0.4105	0.2050	0.029*	

### Atomic displacement parameters (Å^2^)

**Table:**

	*U*^11^	*U*^22^	*U*^33^	*U*^12^	*U*^13^	*U*^23^
P1	0.0158 (2)	0.01588 (19)	0.01475 (19)	0.00488 (14)	0.00474 (14)	0.00582 (14)
O1	0.0200 (5)	0.0226 (5)	0.0143 (5)	0.0084 (4)	0.0057 (4)	0.0046 (4)
O2	0.0203 (5)	0.0268 (6)	0.0200 (5)	0.0101 (4)	0.0074 (4)	0.0050 (4)
O3	0.0249 (5)	0.0174 (5)	0.0173 (5)	0.0062 (4)	0.0046 (4)	0.0023 (4)
C1	0.0199 (7)	0.0190 (7)	0.0177 (7)	0.0069 (6)	0.0072 (6)	0.0094 (6)
C2	0.0261 (8)	0.0239 (7)	0.0157 (7)	0.0079 (6)	0.0065 (6)	0.0063 (6)
C3	0.0247 (8)	0.0312 (8)	0.0177 (7)	0.0083 (7)	0.0023 (6)	0.0103 (6)
C4	0.0246 (8)	0.0338 (9)	0.0258 (8)	0.0154 (7)	0.0077 (6)	0.0144 (7)
C5	0.0282 (8)	0.0258 (8)	0.0200 (7)	0.0142 (6)	0.0090 (6)	0.0088 (6)
C6	0.0235 (7)	0.0180 (7)	0.0173 (7)	0.0075 (6)	0.0075 (6)	0.0085 (6)
C7	0.0242 (7)	0.0159 (7)	0.0152 (7)	0.0067 (6)	0.0076 (6)	0.0050 (5)
C8	0.0197 (7)	0.0143 (6)	0.0157 (7)	0.0032 (5)	0.0069 (6)	0.0054 (5)
C9	0.0180 (7)	0.0170 (7)	0.0157 (7)	0.0033 (5)	0.0056 (5)	0.0060 (5)
C10	0.0158 (7)	0.0181 (7)	0.0156 (7)	0.0033 (5)	0.0066 (5)	0.0046 (5)
C11	0.0181 (7)	0.0176 (7)	0.0132 (6)	0.0040 (5)	0.0031 (5)	0.0035 (5)
C12	0.0161 (7)	0.0177 (7)	0.0186 (7)	0.0052 (5)	0.0054 (5)	0.0053 (6)
C13	0.0231 (8)	0.0242 (7)	0.0185 (7)	0.0047 (6)	0.0060 (6)	0.0086 (6)
C14	0.0225 (8)	0.0280 (8)	0.0173 (7)	0.0048 (6)	0.0028 (6)	0.0053 (6)
C15	0.0190 (7)	0.0298 (8)	0.0249 (8)	−0.0010 (6)	0.0044 (6)	0.0050 (7)
C16	0.0247 (8)	0.0379 (9)	0.0256 (8)	−0.0019 (7)	0.0096 (7)	0.0131 (7)
C17	0.0205 (7)	0.0297 (8)	0.0178 (7)	0.0032 (6)	0.0061 (6)	0.0085 (6)
C18	0.0259 (8)	0.0220 (7)	0.0168 (7)	0.0108 (6)	0.0100 (6)	0.0097 (6)
C19	0.0321 (9)	0.0225 (8)	0.0309 (8)	0.0085 (7)	0.0146 (7)	0.0105 (7)
C20	0.0533 (11)	0.0227 (8)	0.0363 (9)	0.0135 (8)	0.0247 (9)	0.0128 (7)
C21	0.0681 (13)	0.0321 (9)	0.0290 (9)	0.0317 (9)	0.0267 (9)	0.0174 (8)
C22	0.0452 (10)	0.0527 (11)	0.0235 (8)	0.0361 (9)	0.0151 (8)	0.0183 (8)
C23	0.0284 (8)	0.0332 (9)	0.0182 (7)	0.0155 (7)	0.0069 (6)	0.0089 (6)
C24	0.0163 (7)	0.0184 (7)	0.0158 (7)	0.0014 (5)	0.0040 (5)	0.0041 (5)
C25	0.0320 (9)	0.0336 (9)	0.0297 (8)	0.0166 (7)	0.0170 (7)	0.0182 (7)
C26	0.0362 (10)	0.0445 (10)	0.0385 (10)	0.0236 (8)	0.0228 (8)	0.0197 (8)
C27	0.0292 (9)	0.0338 (9)	0.0253 (8)	0.0026 (7)	0.0161 (7)	0.0055 (7)
C28	0.0396 (9)	0.0213 (8)	0.0221 (8)	−0.0013 (7)	0.0142 (7)	0.0057 (6)
C29	0.0306 (8)	0.0188 (7)	0.0211 (7)	0.0048 (6)	0.0091 (6)	0.0067 (6)

### Geometric parameters (Å, º)

**Table:**

P1—C11	1.7237 (14)	C14—C15	1.384 (2)
P1—C12	1.8014 (15)	C14—H14A	0.9500
P1—C18	1.8019 (15)	C15—C16	1.389 (2)
P1—C24	1.8171 (15)	C15—H15A	0.9500
O1—C1	1.3769 (17)	C16—C17	1.387 (2)
O1—C9	1.3838 (17)	C16—H16A	0.9500
O2—C9	1.2064 (18)	C17—H17A	0.9500
O3—C10	1.2586 (17)	C18—C23	1.389 (2)
C1—C2	1.386 (2)	C18—C19	1.400 (2)
C1—C6	1.396 (2)	C19—C20	1.385 (2)
C2—C3	1.382 (2)	C19—H19A	0.9500
C2—H2A	0.9500	C20—C21	1.380 (3)
C3—C4	1.397 (2)	C20—H20A	0.9500
C3—H3A	0.9500	C21—C22	1.377 (3)
C4—C5	1.377 (2)	C21—H21A	0.9500
C4—H4A	0.9500	C22—C23	1.395 (2)
C5—C6	1.403 (2)	C22—H22A	0.9500
C5—H5A	0.9500	C23—H23A	0.9500
C6—C7	1.436 (2)	C24—C25	1.391 (2)
C7—C8	1.350 (2)	C24—C29	1.394 (2)
C7—H7A	0.9500	C25—C26	1.390 (2)
C8—C9	1.4610 (19)	C25—H25A	0.9500
C8—C10	1.5123 (19)	C26—C27	1.383 (3)
C10—C11	1.395 (2)	C26—H26A	0.9500
C11—H11A	0.9500	C27—C28	1.378 (3)
C12—C17	1.388 (2)	C27—H27A	0.9500
C12—C13	1.398 (2)	C28—C29	1.390 (2)
C13—C14	1.388 (2)	C28—H28A	0.9500
C13—H13A	0.9500	C29—H29A	0.9500
			
C11—P1—C12	114.64 (7)	C15—C14—H14A	120.1
C11—P1—C18	106.69 (7)	C13—C14—H14A	120.1
C12—P1—C18	108.00 (7)	C14—C15—C16	120.32 (14)
C11—P1—C24	114.32 (7)	C14—C15—H15A	119.8
C12—P1—C24	105.28 (6)	C16—C15—H15A	119.8
C18—P1—C24	107.59 (7)	C17—C16—C15	120.08 (15)
C1—O1—C9	122.54 (11)	C17—C16—H16A	120.0
O1—C1—C2	117.19 (13)	C15—C16—H16A	120.0
O1—C1—C6	120.27 (13)	C16—C17—C12	119.93 (14)
C2—C1—C6	122.53 (13)	C16—C17—H17A	120.0
C3—C2—C1	118.02 (14)	C12—C17—H17A	120.0
C3—C2—H2A	121.0	C23—C18—C19	119.69 (14)
C1—C2—H2A	121.0	C23—C18—P1	122.02 (12)
C2—C3—C4	120.94 (14)	C19—C18—P1	118.27 (12)
C2—C3—H3A	119.5	C20—C19—C18	120.03 (16)
C4—C3—H3A	119.5	C20—C19—H19A	120.0
C5—C4—C3	120.28 (14)	C18—C19—H19A	120.0
C5—C4—H4A	119.9	C21—C20—C19	119.81 (17)
C3—C4—H4A	119.9	C21—C20—H20A	120.1
C4—C5—C6	120.17 (14)	C19—C20—H20A	120.1
C4—C5—H5A	119.9	C22—C21—C20	120.71 (15)
C6—C5—H5A	119.9	C22—C21—H21A	119.6
C1—C6—C5	118.03 (13)	C20—C21—H21A	119.6
C1—C6—C7	117.86 (13)	C21—C22—C23	120.09 (16)
C5—C6—C7	123.85 (13)	C21—C22—H22A	120.0
C8—C7—C6	121.52 (13)	C23—C22—H22A	120.0
C8—C7—H7A	119.2	C18—C23—C22	119.60 (16)
C6—C7—H7A	119.2	C18—C23—H23A	120.2
C7—C8—C9	119.72 (13)	C22—C23—H23A	120.2
C7—C8—C10	118.80 (12)	C25—C24—C29	119.40 (14)
C9—C8—C10	121.38 (12)	C25—C24—P1	117.57 (11)
O2—C9—O1	116.03 (12)	C29—C24—P1	123.02 (11)
O2—C9—C8	126.74 (13)	C26—C25—C24	119.95 (15)
O1—C9—C8	117.16 (12)	C26—C25—H25A	120.0
O3—C10—C11	125.68 (13)	C24—C25—H25A	120.0
O3—C10—C8	117.62 (12)	C27—C26—C25	120.35 (16)
C11—C10—C8	116.45 (12)	C27—C26—H26A	119.8
C10—C11—P1	123.73 (11)	C25—C26—H26A	119.8
C10—C11—H11A	118.1	C28—C27—C26	119.91 (15)
P1—C11—H11A	118.1	C28—C27—H27A	120.0
C17—C12—C13	119.83 (13)	C26—C27—H27A	120.0
C17—C12—P1	119.88 (11)	C27—C28—C29	120.33 (15)
C13—C12—P1	120.23 (11)	C27—C28—H28A	119.8
C14—C13—C12	120.01 (14)	C29—C28—H28A	119.8
C14—C13—H13A	120.0	C28—C29—C24	120.05 (15)
C12—C13—H13A	120.0	C28—C29—H29A	120.0
C15—C14—C13	119.81 (14)	C24—C29—H29A	120.0
			
C9—O1—C1—C2	170.11 (13)	C24—P1—C12—C13	−44.96 (13)
C9—O1—C1—C6	−9.35 (19)	C17—C12—C13—C14	1.6 (2)
O1—C1—C2—C3	−178.61 (13)	P1—C12—C13—C14	−175.62 (12)
C6—C1—C2—C3	0.8 (2)	C12—C13—C14—C15	−1.5 (2)
C1—C2—C3—C4	−1.1 (2)	C13—C14—C15—C16	0.0 (2)
C2—C3—C4—C5	0.3 (2)	C14—C15—C16—C17	1.3 (3)
C3—C4—C5—C6	0.8 (2)	C15—C16—C17—C12	−1.2 (3)
O1—C1—C6—C5	179.69 (12)	C13—C12—C17—C16	−0.3 (2)
C2—C1—C6—C5	0.3 (2)	P1—C12—C17—C16	176.96 (12)
O1—C1—C6—C7	5.4 (2)	C11—P1—C18—C23	−121.10 (13)
C2—C1—C6—C7	−174.07 (13)	C12—P1—C18—C23	2.62 (14)
C4—C5—C6—C1	−1.1 (2)	C24—P1—C18—C23	115.81 (13)
C4—C5—C6—C7	172.88 (14)	C11—P1—C18—C19	57.07 (13)
C1—C6—C7—C8	3.8 (2)	C12—P1—C18—C19	−179.21 (11)
C5—C6—C7—C8	−170.22 (14)	C24—P1—C18—C19	−66.02 (13)
C6—C7—C8—C9	−8.9 (2)	C23—C18—C19—C20	−1.1 (2)
C6—C7—C8—C10	167.60 (13)	P1—C18—C19—C20	−179.28 (12)
C1—O1—C9—O2	−178.77 (12)	C18—C19—C20—C21	−1.4 (2)
C1—O1—C9—C8	4.13 (18)	C19—C20—C21—C22	2.6 (3)
C7—C8—C9—O2	−171.75 (14)	C20—C21—C22—C23	−1.4 (2)
C10—C8—C9—O2	11.9 (2)	C19—C18—C23—C22	2.3 (2)
C7—C8—C9—O1	5.00 (19)	P1—C18—C23—C22	−179.58 (11)
C10—C8—C9—O1	−171.38 (12)	C21—C22—C23—C18	−1.1 (2)
C7—C8—C10—O3	38.62 (19)	C11—P1—C24—C25	47.08 (14)
C9—C8—C10—O3	−144.96 (13)	C12—P1—C24—C25	−79.63 (13)
C7—C8—C10—C11	−135.98 (14)	C18—P1—C24—C25	165.36 (12)
C9—C8—C10—C11	40.43 (18)	C11—P1—C24—C29	−134.07 (12)
O3—C10—C11—P1	−6.8 (2)	C12—P1—C24—C29	99.21 (13)
C8—C10—C11—P1	167.32 (10)	C18—P1—C24—C29	−15.79 (14)
C12—P1—C11—C10	55.66 (14)	C29—C24—C25—C26	0.9 (2)
C18—P1—C11—C10	175.16 (12)	P1—C24—C25—C26	179.75 (13)
C24—P1—C11—C10	−66.05 (14)	C24—C25—C26—C27	−0.4 (3)
C11—P1—C12—C17	11.30 (15)	C25—C26—C27—C28	−0.4 (3)
C18—P1—C12—C17	−107.47 (12)	C26—C27—C28—C29	0.8 (3)
C24—P1—C12—C17	137.81 (12)	C27—C28—C29—C24	−0.3 (2)
C11—P1—C12—C13	−171.47 (11)	C25—C24—C29—C28	−0.5 (2)
C18—P1—C12—C13	69.77 (13)	P1—C24—C29—C28	−179.31 (11)

### Hydrogen-bond geometry (Å, º)

**Table:**

*D*—H···*A*	*D*—H	H···*A*	*D*···*A*	*D*—H···*A*
C2—H2*A*···O2^i^	0.95	2.45	3.378 (2)	166
C7—H7*A*···O3^ii^	0.95	2.28	3.171 (2)	156
C22—H22*A*···O2^iii^	0.95	2.48	3.398 (2)	163
C25—H25*A*···O3	0.95	2.31	3.168 (2)	150
C28—H28*A*···O1^iv^	0.95	2.54	3.281 (2)	135

Symmetry codes: (i) −*x*+1, −*y*+1, −*z*+2; (ii) −*x*+1, −*y*, −*z*+1; (iii) −*x*, −*y*+1, −*z*+1; (iv) −*x*+1, −*y*+1, −*z*+1.
